# 

**Published:** 1906-09-29

**Authors:** 


CONTENTS.

**Payments for Vaccination** - 451

**Sanitary Inspectors and Public Funds** - 452

**Annotations** - 453

**Medical Opinion and Movement** - 454

**The Celebrations of the Quatro-Centenary of Aberdeen** - 455

**The Book World of Medicine and Science** - 461

**Medical Appointments** - 461

**Hospital Clinics—**

The Treatment of Exophthalmic Goitre - 462

Lumbar Puncture - 463

**Hospital Administration—**

The Royal Hospital for Incurables - 464

The British Medical Association in Toronto - 465

The Fitting and Furnishing of Wards and Operating Theatres 466

Trips for Health and Pleasure - 467

*NURSING SECTION.*

**Notes on News from the Nursing World—**

The Nurses' Hostel Board and Miss Hume—The Midwives Act and the Untrained Midwife—Well done, Grantham!—Nurses Censured by a Jury—Night Work and Operations—A Fatal Act of Carelessness—A Curious Situation at Auckland—A Candidate for Registration under the Midwives Act—Memorial to an Army Sister—Nurses' Social Union—Lectures at Huddersfield—Dispensing with the Night Nurse—Vacancies in Queen Alexandra's Military Nursing Service—Cricket Matches and District Nursing—Home for Invalid French Nurses—The Duchess of Norfolk's Association—Aberdeen Eye Institution—Short Items - 365-368

**The Nursing Outlook** - 369

**The Care and Nursing of the Insane** - 370

**The Nurses' Clinic** - 371

**Incidents in a Nurse's Life** - 371

**Some Impressions of my First Operation** - 372

**The Nurses of the Isle of Wight County Hospital** 372

**A Nurse on Health** - 374

**The Nurses' Hostel** - 375

**The Accident to the Scotch Express** - 375

**Everybody's Opinion** - 376

**Appointments** - 376

**Novelties for Nurses** - 376

**A Book and Its Story** - 377

**Notes and Queries** - 378

**For Reading to the Sick** - 378

